# *Ckip-1* 3′-UTR Attenuates Simulated Microgravity-Induced Cardiac Atrophy

**DOI:** 10.3389/fcell.2021.796902

**Published:** 2022-02-02

**Authors:** Yinglong Zhao, Guohui Zhong, Ruikai Du, Dingsheng Zhao, Jianwei Li, Yuheng Li, Wenjuan Xing, Xiaoyan Jin, Wenjuan Zhang, Weijia Sun, Caizhi Liu, Zizhong Liu, Xinxin Yuan, Guanghan Kan, Xuan Han, Qi Li, Yan-Zhong Chang, Yingxian Li, Shukuan Ling

**Affiliations:** ^1^ State Key Laboratory of Space Medicine Fundamentals and Application, China Astronaut Research and Training Center, Beijing, China; ^2^ Key Laboratory of Molecular and Cellular Biology of Ministry of Education, College of Life Science, Hebei Normal University, Shijiazhuang, China; ^3^ School of Aerospace Medicine, Fourth Military Medical University, Xi’an, China; ^4^ State Key Laboratory of Proteomics, National Center of Protein Sciences (Beijing), Beijing Institute of Lifeomics, Beijing, China

**Keywords:** *Ckip-1* 3′-UTR, simulated microgravity, cardiac atrophy, lipid accumulation, CaMKK2

## Abstract

Microgravity prominently affected cardiovascular health, which was the gravity-dependent physical factor. Deep space exploration had been increasing in frequency, but heart function was susceptible to conspicuous damage and cardiac mass declined in weightlessness. Understanding of the etiology of cardiac atrophy exposed to microgravity currently remains limited. The 3′-untranslated region (UTR) of casein kinase-2 interacting protein-1 (*Ckip-1*) was a pivotal mediator in pressure overload-induced cardiac remodeling. However, the role of *Ckip-1* 3′-UTR in the heart during microgravity was unknown. We analyzed *Ckip-1* mRNA 3′-UTR and coding sequence (CDS) expression levels in ground-based analogs such as mice hindlimb unloading (HU) and rhesus monkey head-down bed rest model. *Ckip-1* 3′-UTR had transcribed levels in the opposite change trend with cognate CDS expression in the hearts. We then subjected wild-type (WT) mice and cardiac-specific *Ckip-1* 3′-UTR-overexpressing mice to hindlimb unloading for 28 days. Our results uncovered that *Ckip-1* 3′-UTR remarkably attenuated cardiac dysfunction and mass loss in simulated microgravity environments. Mechanistically, *Ckip-1* 3′-UTR inhibited lipid accumulation and elevated fatty acid oxidation-related gene expression in the hearts through targeting calcium/calmodulin-dependent kinase 2 (CaMKK2) and activation of the AMPK-PPARα-CPT1b signaling pathway. These findings demonstrated *Ckip-1* 3′-UTR was an important regulator in atrophic heart growth after simulated microgravity.

## Introduction

For decades, human space activities are more and more ambitious. Nevertheless, the health risks of the astronaut are a principal hindrance to reliable manned deep-space exploration (Löbrich and Jeggo, 2019; Afshinnekoo et al., 2020). National space agencies urgently need to promote the basic life-sciences studies to attempt to understand these things as soon as possible, which makes increased efforts to evaluate and mitigate this damage for the crew (Foldager et al., 1996; Shi et al., 2021). Relative to the 1 *g* gravity on the Earth, the substantially reduced gravity in the space environment elicits major deconditioning to the physiology of the human body (Dai et al., 2019; Garrett-Bakelman et al., 2019). With all of these physiological effects in microgravity, cardiac health is mechanosensitive and susceptible to gravitational force changes (Tank and Jordan, 2015). Spaceflight reduces venous return to the heart by causing blood to pool in lower body compartments. These hemodynamic effects lead to decrease cardiac stroke volume and insufficient heart muscle constriction, which will eventually result in cardiac atrophy (Hughson et al., 2018). Ground-based models with head-down tilt bed rest and hindlimb unloading (HU) have been used to study the physiological effects of microgravity loads since biological responses are similar (Perhonen et al., 2001; Morey-Holton et al., 2005; Platts et al., 2009). Several physical measurements in astronauts after long-duration and short-duration space missions have revealed attenuated cardiac function and mass. However, very limited is known about the clinical conditions of cardiac atrophy exposure to microgravity.

Ubiquitously expressed 3'-untranslated region (UTR) is the long tail of mRNA molecule, which is contiguous with the upstream protein-coding sequence (CDS) (Keene, 2007). Regulatory signals within 3′-UTR can affect mRNA localization, stability, and translational efficiency through interactions with proteins and microRNAs. Genetic variations at 3′-UTR are associated with various disease processes, which cardiological phenotypes are pronounced. These mRNA 3′-UTR traits imply that between proteome and genome still exists an attractive and mysterious world, with its own set of dimensions and rules (Conne et al., 2000; Orengo et al., 2008; Mayr and Bartel, 2009). Interestingly, recent studies revealed a discrepant expression of the cognate 3′-UTR and CDS in mammals and bacteria (Mercer et al., 2011; Chao and Vogel, 2016; Malka et al., 2017). Many genes showed high expression of the 3′-UTR regions in the presence of low expression of CDS regions, but others exhibited the opposite in neurons (Kocabas et al., 2015). In particular, our previous works found widespread unbalanced expression levels of 3′-UTR and their cognate coding sequence in heart failure patients, in which casein kinase 2 interacting protein-1 (*Ckip-1*) is the most prominent (Bernasconi and Kuster, 2021; Zhao et al., 2021). CKIP-1 protein regulates the HDAC4/MEF2 pathway and suppresses cardiac hypertrophy (Ling et al., 2012). In cardiac-specific *Ckip-1* 3′-UTR overexpression under *Ckip-1* knockout background (*Ckip-1* KO/3′-UTR TG) mice, *Ckip-1* 3′-UTR inhibits pathological cardiac hypertrophy independently of its cognate protein by fatty acid metabolism pathway (Zhao et al., 2021). Moreover, CKIP-1 protein protects against the development of cardiac atrophy in mice tail suspension models (Ling et al., 2018). The potential role of *Ckip-1* 3′-UTR in the microgravity-induced atrophic heart is unknown.

Cardiac deconditioning in a microgravity environment drives the change of various underlying molecular mechanisms. In the past, we identified HDAC4, ERK1/2, AMP-activated protein kinase (AMPK), LC3-II, and intracellular secondary messenger Ca^2+^ are pivotally involved in cardiac microgravity phenotype (Zhong et al., 2016; Liu et al., 2020). The heart consumes plenty of ATP for maintaining contraction. The integration of metabolism pathways contributes to supporting cardiac homeostasis, but microgravity tends to be decreased in metabolic demand (Herault et al., 2000; Goswami et al., 2019; da Silveira et al., 2020; Gallo et al., 2020). The National Aeronautics and Space Administration (NASA) reported the transcriptomic analysis of *Drosophila* hearts from the International Space Station (ISS) compared with 1*g* controls. The results showed that carbohydrate metabolic processes are downregulated in response to microgravity (Walls et al., 2020). Another study found that HU rats impaired glucose utilization in the heart (Wang et al., 2020). Due to the limited cardiac atrophy research, the metabolism pathway of heart tissues under microgravity is poorly understood.

Our results showed *Ckip-1* 3′-UTR expression is different from the corresponding coding region of the same strand in the heart from mice and rhesus monkeys during microgravity. Cardiac overexpression of *Ckip-1* 3′-UTR region alleviates cardiac atrophy in tail-suspended mice, thereby improving heart function and increasing cardiac efficiency. *Ckip-1* 3′-UTR is involved in the anti-atrophic action of AMPK, inhibiting lipid accumulation and facilitating ATP supply in the heart. Here, we reported that *Ckip-1* 3′-UTR functions as a crucial long non-coding RNA (lncRNA) to regulate cardiac atrophy by targeting the fatty acid metabolism signaling pathway.

## Materials and Methods

### Mouse and Rhesus Monkey Data

This study was approved by the Ethics Committee of the China Astronaut Research and Training Center with the permit number ACC-IACUC-2020-002. All the procedures performed in mice and rhesus monkeys complied with the National Institutes of Health Guidelines on the Use of Laboratory Animals. Cardiac-specific *Ckip-1* 3′-UTR transgenic mice (3′-UTR TG) had been obtained in previous reports (Zhao et al., 2021).

The 2-month-old male 3′-UTR TG mice and age-matched littermate wild-type (WT) mice were subjected to tail suspension for 28 days. Simulation of microgravity was done by mechanically unloading mouse hindlimbs using a method described previously. Each one of these mice was breeding in separate cage and accessed food and water freely. The hindlimbs of mice were suspended on the air, and reaction forces from the ground were prevented, but forelimbs can move on the ground. Control mice were also maintained for 28 days in this system without tail suspension.

The 4–8-year-old rhesus monkeys were purchased from Beijing Xieerxin Biology Resource (Beijing, China). The rhesus monkeys performed head-down bed rest for 42 days and held a 10° inclined angle to the ground, as previously described (Chen et al., 2016). We monitored real-time the emotion and health of these rhesus monkeys.

### Echocardiography

To perform transthoracic echocardiography assay, mice were continuously anesthetized with 2% isoflurane and 95% oxygen. Two-dimensional guided M-mode echocardiography was conducted using a high-resolution imaging system (Vevo 1100, Visual-Sonics Inc., Toronto, Canada), recording images in parasternal long- and short-axis views. Left ventricular fractional shortening, ejection fraction, chamber diameter, and wall thickness in M-mode images were measured. Left ventricular (LV) cavity size and wall thickness are measured in at least three beats from each projection. Averaged LV wall thickness [interventricular septum (IVS) and posterior wall (PW) thickness] and internal dimensions at diastole and systole (LVIDd and LVIDs, respectively) are measured. LV fractional shortening [(LVIDd − LVIDs)/LVIDd], relative wall thickness [(IVS thickness + PW thickness)/LVIDd], and LV mass [LV mass = 1.053 × (LVID; *d* + LVPW; *d* + IVS; d3 − LVID; d3)] are calculated from the M-mode measurements. LV ejection fraction (EF) was calculated from the LV cross-sectional area (2-D short-axis view) using the equation LV %EF = (LV Vol; *d* − LV Vol; *s*)/LV Vol; *d* × 100%. Blinding procedures were carried out in the echocardiograph analysis of these mice.

### Histological Analysis

Mouse hearts were removed and fixed in 4% paraformaldehyde (pH 7.4) for 24 h. Heart section with hematoxylin and eosin (H&E) and Masson’s trichrome staining were obtained from paraffin-embedded tissue block. Frozen sections were applied to stain cardiomyocyte membranes by wheat germ agglutinin (WGA) for measurement of cell size (Sigma-Aldrich, 61767). Cross sections of the left ventricle from the middle section were stained to determine the cross-sectional area (CSA) of cardiomyocytes. About 12 cross-sectioned cardiomyocytes were counted in at least three images obtained from each left ventricle. Only round to ovoid cells with visible round nucleus were considered for CSA measurements. Each cell was individually traced, and its CSA was directly determined. For lipid content examination, oil red O (ORO) staining in heart frozen sections was used to evaluate lipid accumulation, and heart lipid droplets (LD) were assessed with BODIPY™ 493/503 (20 μM) probes (Invitrogen, United States). Images were observed with a fluorescence microscope and confocal microscopy. Confocal imaging was performed with an LSM710 microscope (Zeiss) with a ×40, 1.3 NA oil immersion objective, and the pinhole was nominally set for a 1-μm optical section. Fluo-4 AM was excited at 488 nm, and fluorescence emission was measured at 490–550 nm. The quantity of cardiomyocyte area and ORO staining were analyzed by ImageJ software (NIH).

### Total ATP Measurement

Total ATP was extracted and lysed with cold trichloroacetic acid from mouse heart tissue. ATP levels were normalized to protein content, subsequently detected with the ATP Assay Kit (Promega) according to the manufacturer’s instructions.

### Western Blot

Heart tissues were lysed in cold RIPA buffer: NaCl 150; Tris-HCl 50 (pH 7.4); EDTA 2; and supplemented with 1% NP-40, 0.1% SDS, 1% sodium deoxycholate, and 1x protease inhibitor cocktail (Promega, United States). Protein samples were separated in 10% SDS-PAGE gel (30% acr-bis, 10% SDS, 1 M Tris-Hcl, TEMED, and 10% ammonium persulfate) and immediately transferred to PVDF membranes (BioRad, United States). Membranes were blocked with ×1 TBST supplemented with 5% skim milk for 1 h and incubated with primary antibodies overnight at 4°C. The antibodies used in western blots are as follows: calcium/calmodulin-dependent kinase 2 (CaMKK2) (1:1,000, ABclonal, A9899), AMPK (1:1,000, Cell Signaling Technology, 2532S), *p*-AMPK (1:1,000, T172) (Cell Signaling Technology, 2535S), peroxisome proliferator-activated receptor α (PPARα) (1:1,000, ABclonal, A3123), CPT1b (1:1,000, ABclonal, A6796), and *α*-tubulin (1:1,000, Sigma, T9026). Blots were incubated with horseradish peroxidase-conjugated goat anti-mouse or anti-rabbit secondary antibodies (ZSGB-Bio; cat. ZB-2305 and ZB-2301). Visualization used ECL detection reagent (Millipore). The intensities of bands were analyzed with ImageJ software (NIH).

### RNA Extraction and Real-Time PCR

RNA was extracted from heart tissue with TRIzol (Invitrogen) according to the manufacturer’s instruction. Reverse transcription of RNA samples used the Superscript First-Strand Synthesis Kit (Takara). Quantitative real-time PCR was performed using SYBR green (Takara). The mRNA levels were normalized to *Gapdh* or *18S rRNA* levels. The primers used to analyze mRNA levels are found in Table 1.

**TABLE 1:** Primer sequences.

Species	Gene	Forward primer	Reverse primer
Rhesus monkeys	*Gapdh*	AGC​CCC​ATC​ACC​ATC​TTC​C	AAT​GAG​CCC​CAG​CCT​TCT​C
Rhesus monkeys	*Ckip-1* 3′-UTR	GGCGGGGTGGGGTCT	AAA​AAG​CTT​TAT​CCA​GCC​ACA​CG
Rhesus monkeys	*Ckip-1 CDS*	AAG​AAC​AAT​TCC​GCC​AAG​CG	GTA​GAG​CTG​GTC​CCC​TTT​CA
Mice	*Gapdh*	ACT​CCA​CTC​ACG​GCA​AAT​TCA	GGC​CTC​ACC​CCA​TTT​GAT​G
Mice	*18S rRNA*	GTA​ACC​CGT​TGA​ACC​CCA​TT	CCA​TCC​AAT​CGG​TAG​TAG​CG
Mice	*Ckip-1* 3′-UTR	GGGGGCAGGTCTGAAAT	TGC​AAC​ATT​TGG​AGA​TAA​AGA​G
Mice	*Ckip-1 CDS*	CCG​GAT​GGA​AAC​CAT​CAG​TCT	TCA​GCA​CCA​CAT​AGC​GGT​TT
Mice	*Bnp*	TGT​TTC​TGC​TTT​TCC​TTT​ATC​TG	TCT​TTT​TGG​GTG​TTC​TTT​TGT​GA
Mice	*Col1a1*	CTG​ACT​GGA​AGA​GCG​GAG​AGT	AGA​CGG​CTG​AGT​AGG​GAA​CAC
Mice	*Col3a1*	ACG​TAA​GCA​CTG​GTG​GAC​AG	CAG​GAG​GGC​CAT​AGC​TGA​AC
Mice	*Acox1*	CAC​CCG​TCC​CAA​GAA​CTC​CAG​ATA	AAG​GCA​TGT​AAC​CCG​TAG​CAC​TCC
Mice	*Cpt-1b*	CAT​GTA​TCG​CCG​CAA​ACT​GG	CCT​GGG​ATG​CGT​GTA​GTG​TT

### Statistical Analysis

For statistical analysis, all quantitative data are presented as the mean ± SD. Statistical analysis for comparison of two groups was performed using two-tailed unpaired Student’s *t*-test. Statistical differences among groups were analyzed by one-way or two-way analysis of variance (ANOVA) with a *post hoc* test to determine group differences. All statistical analyses were performed with the Prism software (GraphPad Prism for Windows, version 9.0, Nashville, United States) and SPSS (version 14.0). Differences were considered significant at *p* < .05.

## Results

### Differential Expression of *Ckip-1* 3'-UTR and CDS in Hearts From Mice and Rhesus Monkeys Under Microgravity

**FIGURE 1:**
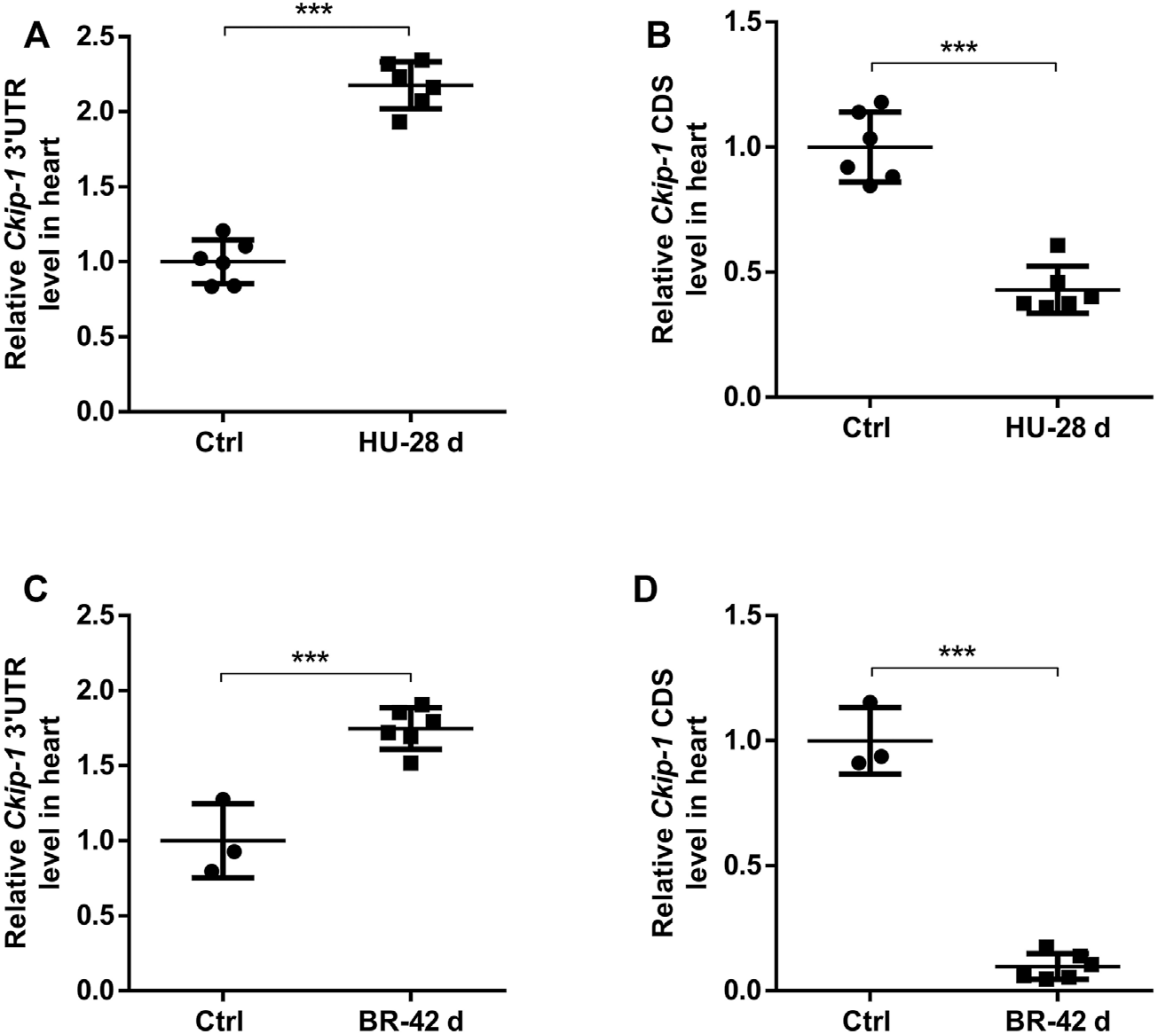
*Ckip-1* 3′-UTR and coding sequence had unbalanced expression in cardiac tissues of mice and rhesus monkeys exposed to simulated microgravity. **(A,B)** Schematic representation of 28-day tail suspension simulated microgravity-induced cardiac atrophy model in mice and the expression of *Ckip-1* 3′-UTR and CDS sequence mRNA levels in hearts from HU-mice and control group (*n* = 6 per group). **(C,D)** A scheme depicting the head-down tilt bed rest model in rhesus monkeys and the real-time PCR analysis of *Ckip-1* 3′-UTR and CDS sequence mRNA levels in the rhesus monkey hearts during simulated microgravity (*n* = 3 for ctrl; *n* = 6 for BR). Data represent the means ± SEM. ***p < .001. Statistical differences between two groups were determined by the unpaired two-tailed Student’s *t*-test. *Ckip-1*, casein kinase-2 interacting protein-1; 3′-UTR, 3′-untranslated region; CDS, coding sequence; HU, hindlimb unloading; BR, bed rest; ctrl, control.

We were interested in understanding how the expression of 3′-UTR and CDS of *Ckip-1* mRNA were altered during cardiac atrophy, since we previously reported *Ckip-1* was a striking gene within the incongruous expression of mRNA 3′-UTR and CDS after heart failure (Zhao et al., 2021). To this end, we subjected wild-type mice to a hindlimb unloading model for simulated microgravity-induced cardiac remodeling. Compared with the control group, *Ckip-1* 3′-UTR expression levels were significantly higher, but *Ckip-1* CDS regions had lower expression levels in the hearts of WT mice after cardiac atrophy (Figures 1A,B). Moreover, we used a physiologically relevant bed rest model of atrophy in the rhesus monkey hearts and detected *Ckip-1* 3′-UTR as well as coding region expression levels. Similarly, simulated microgravity induced upregulation of *Ckip-1* 3′-UTR expression, and *Ckip-1* CDS was expressed in the opposite tendency from its cognate 3′-UTR (Figures 1C,D). Based on the above *Ckip-1* 3′-UTR and CDS region properties and our previous report that CKIP-1 protein was an inhibitor of cardiac remodeling induced by simulated microgravity (Ling et al., 2018), these findings implied that *Ckip-1* 3′-UTR may play a critical role in the development of cardiac atrophy.

### Overexpression of *Ckip-1* 3'-UTR in the Heart Suppressed Simulated Microgravity-Induced Cardiac Remodeling

**FIGURE 2:**
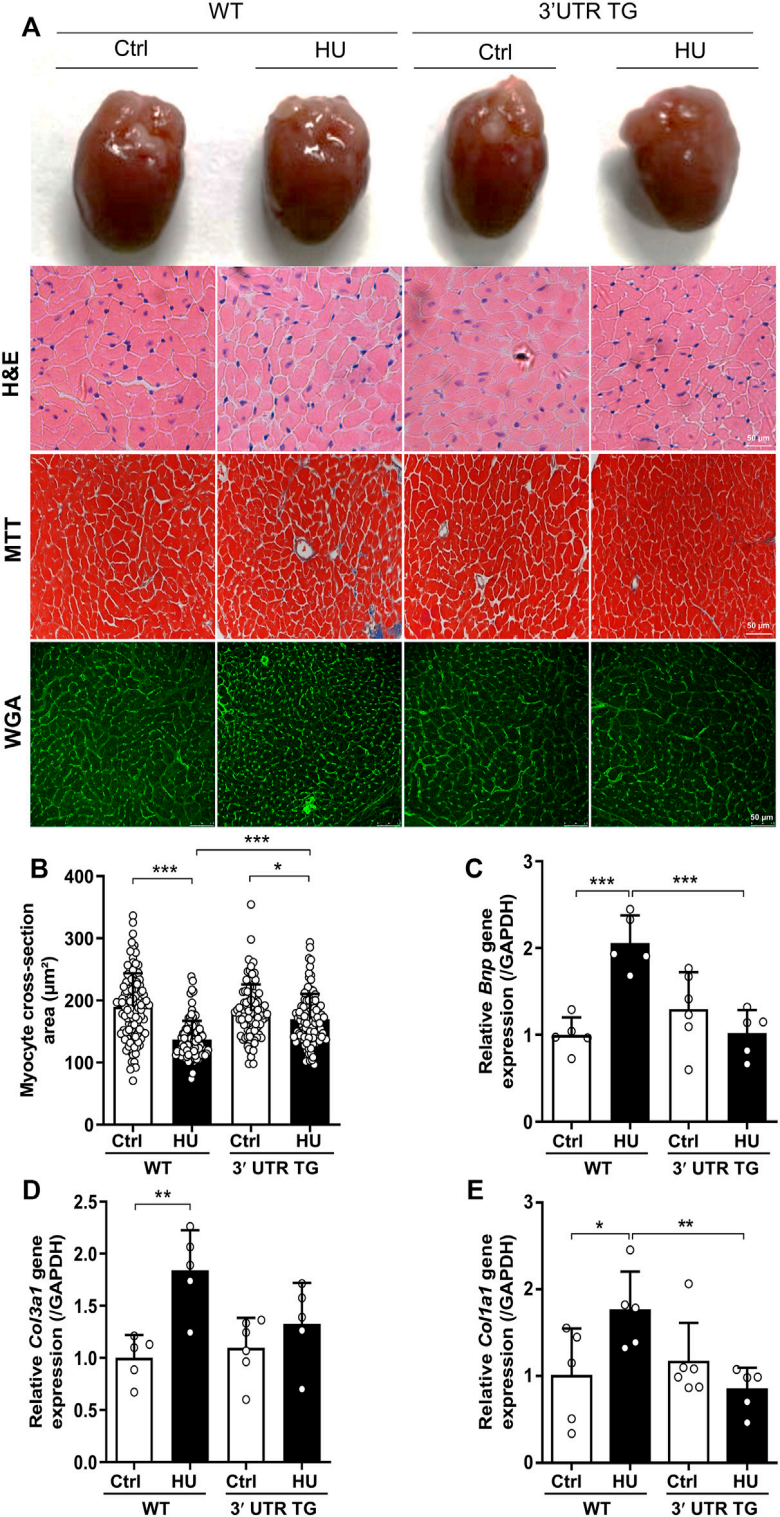
Cardiac-specific Ckip-1 3′-UTR overexpression attenuated simulated microgravity-induced cardiac remodeling in mice. **(A)** Representative images of whole heart and H&E-stained cross sections of littermates WT and 3′-UTR TG mice at 28 days after ctrl and HU operation. Masson trichrome (MTT) and wheat germ agglutinin (WGA) staining. Scale bar in sections, 50 µm. **(B)** Quantification analysis of the cross-sectional area of the cardiomyocytes in 3′-UTR TG and WT mice under simulated microgravity. **(C–E)** Transcript levels of the pathological remodeling marker genes (*Bnp*, *Col3a1*, and *Col1a1*) in heart tissue from the indicated groups (*n* = 5–6). Data represent the means ± SEM. *p < .05, **p < .01, ***p < .001. Statistical differences among groups were analyzed by two-way analysis of variance (ANOVA) followed by the Bonferroni procedure. *Ckip-1*, casein kinase-2 interacting protein-1; 3′-UTR, 3′-untranslated region; HU, hindlimb unloading; H&E, hematoxylin and eosin; TG, transgenic; WT, wild type; Bnp, brain natriuretic peptide; Col3a1, collagen type III alpha 1 chain; Col1a1, collagen type I alpha 1 chain.

To determine the roles of *Ckip-1* 3′-UTR on the heart under microgravity environment, cardiac-specific *Ckip-1* 3′-UTR-overexpressing mice (3′-UTR TG) were generated by using *α*-MHC promoter in cardiomyocytes. Both 3′-UTR TG and wild-type littermate control mice were employed a HU model to induce cardiac atrophy. We carried out histological analysis to evaluate cardiac remodeling and fibrosis in heart tissues. In comparison with the control mice, HU for 4 weeks resulted in a reduction in the global heart size and cardiomyocyte cross-sectional area in WT mice, while 3′-UTR TG mice attenuated the development of atrophic heart upon microgravity (Figures 2A,B). The deeper staining of collagen in heart tissues induced by simulated microgravity was significantly reduced in the overexpression of 3′-UTR TG (Figure 2A). Moreover, we performed reverse transcription polymerase chain reaction (RT-PCR) assay, showing that 3′-UTR TG inhibited the HU-induced upregulation of pathological cardiac remodeling gene expression such as brain natriuretic peptide (*Bnp*), collagen type III alpha 1 chain (*Col3a1*), and collagen type I alpha 1 chain (*Col1a1*) (Figures 2C–E). Our data demonstrated that *Ckip-1* 3′-UTR protected against HU-induced pathological cardiac remodeling.

### *Ckip-1* 3′-UTR Inhibited Cardiac Dysfunction in Mice Exposed to Simulated Microgravity

**FIGURE 3:**
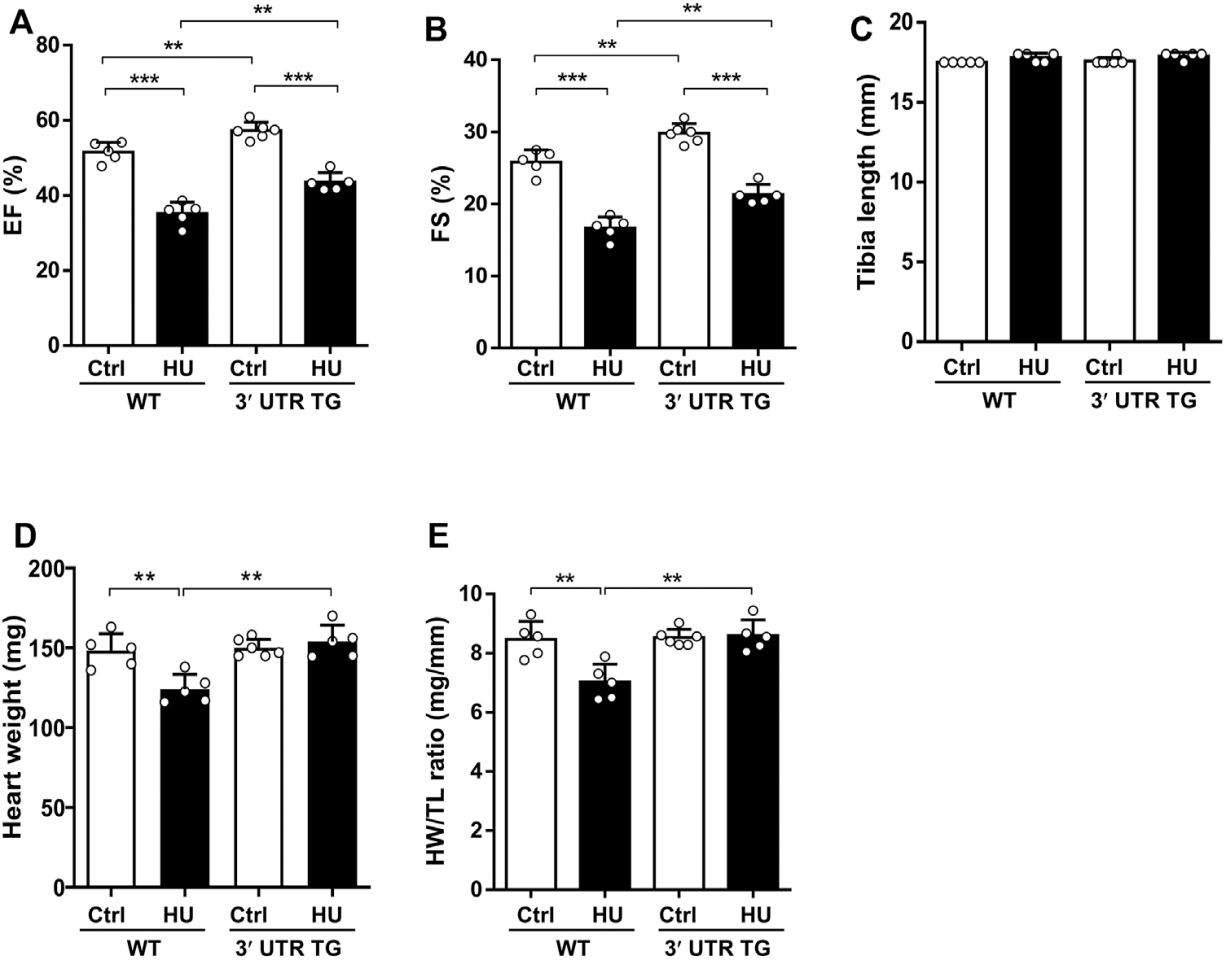
Overexpression of Ckip-1 3′-UTR in the heart improved cardiac function after simulated microgravity. **(A,B)** Echocardiographic analysis of left ventricle ejection fraction (EF) and fractional shortening (FS) from WT and 3′-UTR TG mice in tail suspension and ctrl group. **(C)** Measurements of tibia length in the indicated groups. **(D,E)** Calculated heart weight and heart weight-to-body weight ratios in WT and 3′-UTR TG mice after 28 days of tail suspension. *N* = 5–6 per group. Data represent the means ± SEM. **p < .01, ***p < .001. Statistical differences among groups were analyzed by two-way analysis of variance (ANOVA) followed by the Bonferroni procedure. *Ckip-1*, casein kinase-2 interacting protein-1; 3′-UTR, 3′-untranslated region; TG, transgenic; WT, wild type; ctrl, control.

To monitor the action of *Ckip-1* 3′-UTR in cardiac function under simulated microgravity, we performed echocardiographic measurements and anatomical analysis in the heart from WT and 3′-UTR TG mice following simulated microgravity exposure. The results showed left ventricular EF (Figure 3A) and fractional shortening (FS) (Figure 3B) were remarkably elevated at baseline after *Ckip-1* 3′-UTR overexpression. Four weeks after hindlimb unloading, *Ckip-1* 3′-UTR protected against the deterioration of cardiac function compared with WT mice (Figures 3A,B). There was no significant difference in the tibia length (TL) of these mice (Figure 3C). However, heart weight (HW) and heart weight-to-tibia length ratio (HW/TL) observed in WT mice showed a prominent decrease after simulated microgravity, and *Ckip-1* 3′-UTR overexpression reversed the atrophic effect in the hearts of HU mice (Figures 3D,E). Altogether, these results indicated that *Ckip-1* 3′-UTR prevented the decline of cardiac function and the atrophic pathology induced by simulated microgravity.

### *Ckip-1* 3′-UTR Drove Cardiac Structural Changes After Pathological Atrophic Stimuli

**FIGURE 4:**
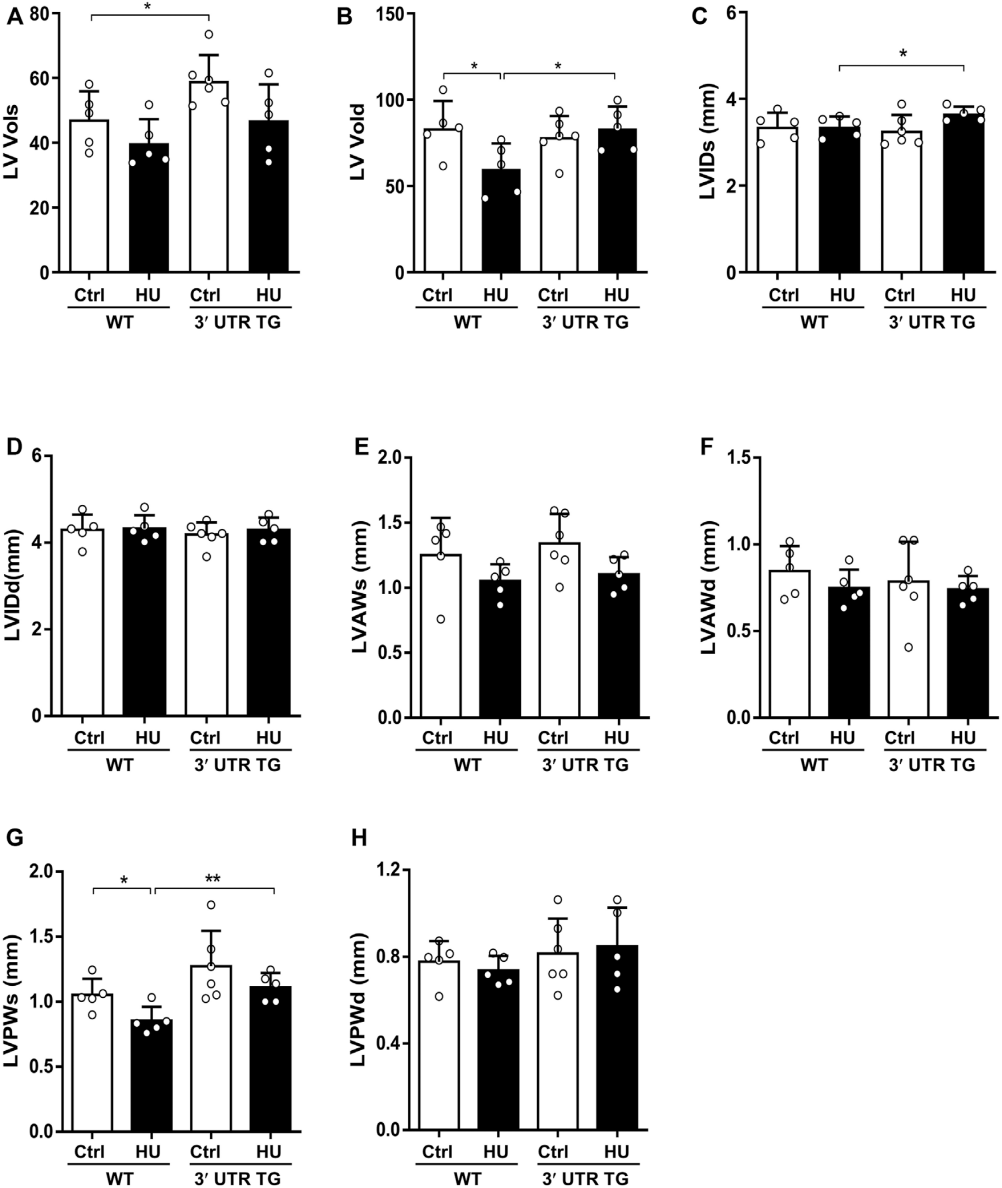
*Ckip-1* 3′-UTR altered cardiac left ventricle structure in response to simulated microgravity. **(A,B)** Echocardiographic measurements of the end-systolic left ventricular volume (LV Vols) and the end-diastolic left ventricular volume (LV Vold) in WT and 3′-UTR TG mice after ctrl and 28-day HU. **(C,D)** Quantitative analysis of the diastolic and systolic left ventricular internal diameter (LVIDd and LVIDs, respectively) in the indicated groups. **(E–H)** The diastolic and systolic left ventricular posterior wall thickness (LVPWd and LVPWs, respectively) and anterior wall thickness (LVAWd and LVAWs, respectively) by echocardiography. *N* = 5–6 per group. Data represent the means ± SEM. **p < .01, ***p < .001. Statistical differences among groups were analyzed by two-way analysis of variance (ANOVA) followed by the Bonferroni procedure. *Ckip-1*, casein kinase-2 interacting protein-1; 3′-UTR, 3′-untranslated region; TG, transgenic; WT, wild type; ctrl, control; HU, hindlimb unloading.

To further interrogate the consequences of *Ckip-1* 3′-UTR overexpression on left ventricular structure in response to hindlimb unloading, we compared the echocardiographic structural parameters of each study group. *Ckip-1* 3′-UTR overexpression can increase the end-systolic left ventricular volume (LV Vols) at baseline, and a trend toward augmented LV Vols was seen with simulated microgravity (Figure 4A). The end-diastolic left ventricular volume (LV Vold) was significantly reduced in HU mice compared with the hearts of control mice, while it exhibited a significant increase in response to simulated microgravity in the hearts from 3′-UTR TG mice (Figure 4B). Measurements of heart end-diastolic left ventricular internal diameter (LVIDs) showed a significant increase in 3′-UTR TG mice after simulated microgravity (Figure 4C). *Ckip-1* 3′-UTR showed little effects on hindlimb unloading-induced end-systolic left ventricular internal diameter (LVIDd) (Figure 4D). *Ckip-1* 3′-UTR overexpression did not alter the end-systolic left ventricular anterior wall thickness (LVAWs) and the end-diastolic left ventricular anterior wall thickness (LVAWd) during simulated microgravity (Figures 4E,F). Exposure to hindlimb unloading resulted in a remarkable reduction in end-systolic left ventricular posterior wall thickness (LVPWs), but *Ckip-1* 3′-UTR overexpression abolished this decline in hearts (Figure 4G). The end-diastolic left ventricular posterior wall thickness (LVPWd) has a similar trend, which was not statistically significant (Figure 4H). These data suggested that *Ckip-1* 3′-UTR overexpression in cardiomyocytes principally inhibited heart contraction disorder in simulated microgravity conditions.

### *Ckip-1* 3′-UTR Restricted Cardiac Lipid Accumulation During Simulated Microgravity

**FIGURE 5:**
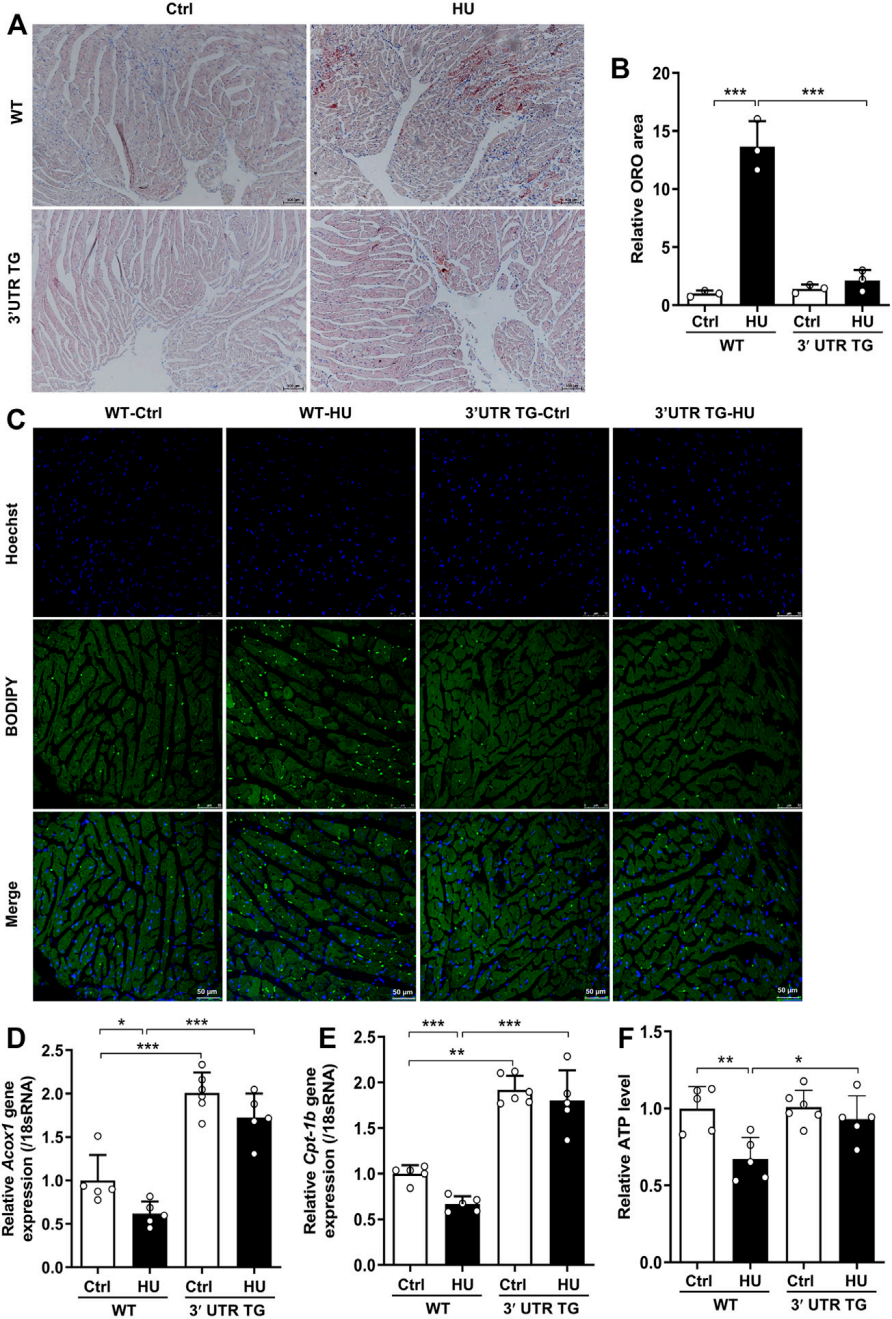
*Ckip-1* 3′-UTR prevented lipid deposition in mice under simulated microgravity. **(A)** Intramyocardial lipid accumulation in the hearts from WT and 3′-UTR TG mice following hindlimb unloading for 28 days. Scale bar in sections, 100 µm. **(B)** Quantification of myocardial oil red O staining (ORO, *n* = 3). **(C)** Representative images of lipid droplet sections in the hearts. Scale bar in sections, 50 µm. **(D,E)** mRNA expression related to fatty acid oxidation enzyme genes in the indicated groups (*n* = 5–6). **(F)** Intracellular ATP content of WT and 3′-UTR TG mice with simulated microgravity (*n* = 5–6). Data represent the means ± SEM. **p < .01, ***p < .001. Statistical differences among groups were analyzed by two-way analysis of variance (ANOVA) followed by the Bonferroni procedure. *Ckip-1*, casein kinase-2 interacting protein-1; 3′-UTR, 3′-untranslated region; TG, transgenic; WT, wild type.

Fatty acids (FAs) are major energy substrates for cardiomyocyte ATP generation, while lipid accumulation in the hearts can evoke cardiac dysfunction (Goldberg et al., 2012; Ritterhoff and Tian, 2017). To assess FAs metabolism in WT mice and 3′-UTR TG mice during cardiac atrophy development, we measured lipid accumulation in the hearts by ORO staining (Figure 5A). In WT hearts, the morphologic analysis revealed that lipid accumulation can be observed after simulated microgravity, and *Ckip-1* 3′-UTR overexpression significantly inhibited this phenotype (Figures 5A,B). In addition, hindlimb unloading induced cardiac LD accumulation compared with the control in WT mice, but less LD number was seen in the heart of 3′-UTR TG mice under pathological atrophic stimuli (Figure 5C). Consistent with mice having this phenotype, LD was deposited in the hearts of rhesus monkeys after bed rest (Supplementary Figure S1). We then examined the cardiac mRNA levels of several genes in the regulation of FA oxidation, including carnitine palmitoyltransferase-1b (*Cpt-1b*) and acyl-coenzyme A oxidase 1 (*Acox1*). The results showed that 3′-UTR TG mice increased the expression of *Cpt-1b* and *Acox1* at baseline, and the decreased expression of *Cpt-1b* and *Acox1* caused by HU stimuli was rescued by the overexpression of *Ckip-1* 3′-UTR (Figures 5D,E). These data confirmed that *Ckip-1* 3′-UTR obviously inhibited abnormal lipid retention and facilitated fatty acid oxidation for ATP generation in the heart with microgravity exposure.

### *Ckip-1* 3′-UTR Exerted Cardioprotective Effects Through the CaKMM2-AMPK-PPARα-CPT1b Axis After Simulated Microgravity

**FIGURE 6:**
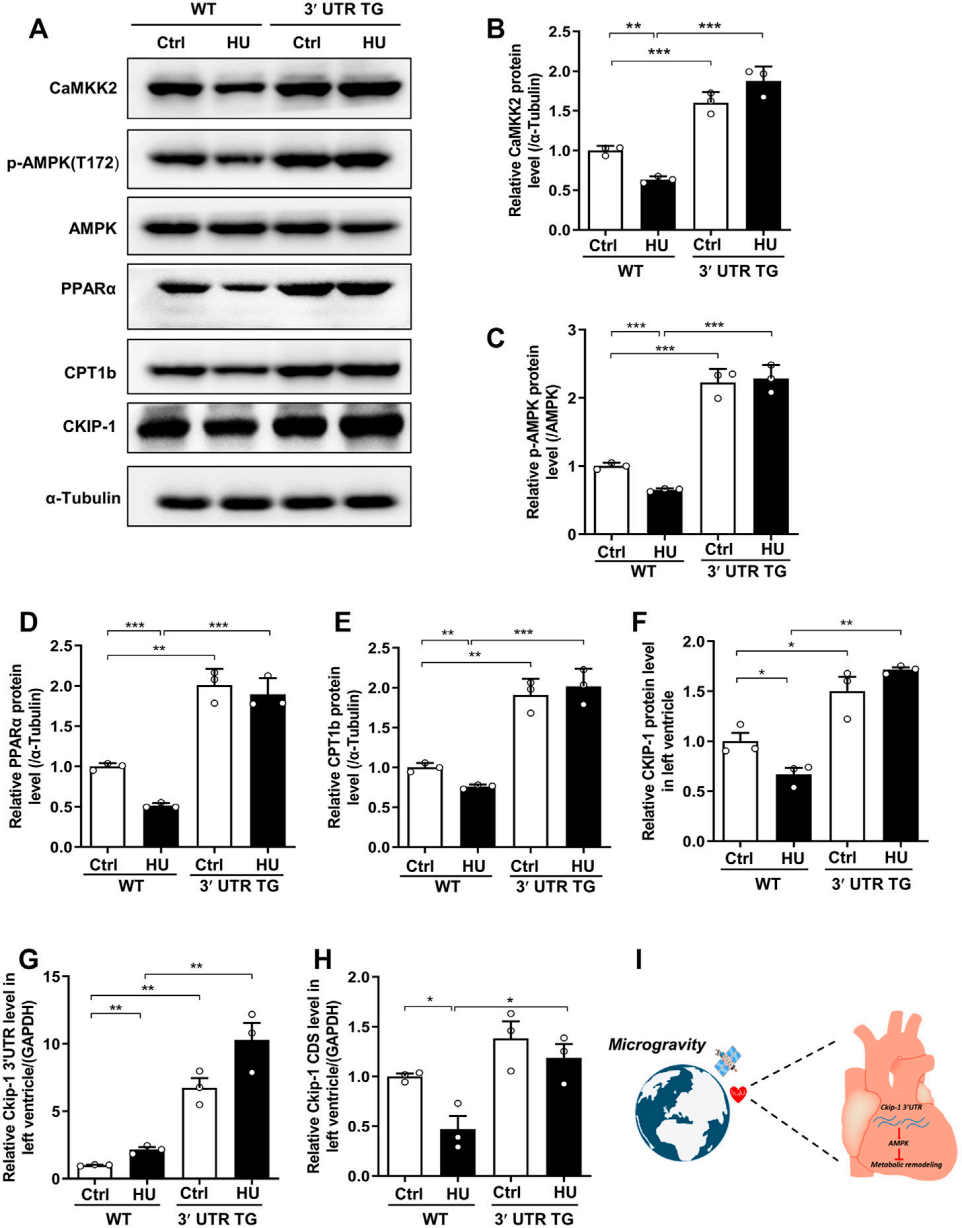
*Ckip-1* 3′-UTR protected against cardiac remodeling during simulated microgravity by activation the CaKMM2-AMPK-PPARα-CPT1b axis. **(A)** Western blot showing the data of CaMKK2, phosphorylated AMPK (T172), AMPK, PPARα, CPT1b, and CKIP-1 in hearts from WT and 3′-UTR TG mice after simulated microgravity. **(B–F)** Quantification of CaMKK2, *p*-AMPK (T172), PPARα, CPT1b, and CKIP-1 levels in WT and 3′-UTR TG mice in response to pathological atrophic stimuli. **(G,H)** qPCR detection of *Ckip-1* 3′-UTR and *Ckip-1* mRNA expression in heart tissues from WT and 3′-UTR TG mice in normal and HU conditions (*n* = 3 for each group). **(I)** The working model showed *Ckip-1* 3′-UTR inhibited simulated microgravity-induced cardiac remodeling through activation of AMPK and ATP supply. Data represent the means ± SEM. ***p < .01, ***p < .001. Statistical differences among groups were analyzed by two-way analysis of variance (ANOVA) followed by the Bonferroni procedure. *Ckip-1*, casein kinase-2 interacting protein-1; 3′-UTR, 3′-untranslated region; TG, transgenic; WT, wild type; AMPK, AMP-activated protein kinase; CaMKK2, calcium/calmodulin-dependent kinase 2; PPARα, peroxisome proliferator-activated receptor α; CPT1b, carnitine palmitoyltransferase1b.

To illustrate the potential mechanism of *Ckip-1* 3′-UTR overexpression on the anti-atrophic role in the heart during simulated microgravity, we examined the proteins related to fatty acid metabolism, which included CaMKK2, AMPK, PPARα, Cpt-1b, and CKIP-1 (Figure 6A). Our previous work proved *Ckip-1* 3′-UTR functioned as a ceRNA to regulate CaMKK2 in the heart. We consistently found that *Ckip-1* 3′-UTR overexpression induced an increase in CaMKK2 protein levels in control and HU mice, while CaMKK2 protein significantly reduced in the heart from WT mice after simulated microgravity (Figures 6A,B). Its downstream crucial energy sensor AMPK, FA metabolism transcription factor PPARα, and key rate-limiting enzyme CPT1b of FA utilization were rescued in 3′-UTR TG mice in response to simulated microgravity. In addition, Western blot analysis showed that CKIP-1 protein levels were increased in the hearts from mice after 3′-UTR overexpression (Figures 6C–F). We then detected the changes of *Ckip-1* 3′-UTR and *Ckip-1* CDS mRNA in heart tissues from WT and 3′-UTR TG mice in normal and HU conditions. The results showed that both the *Ckip-1* 3′-UTR and *Ckip-1* mRNA levels have no significant difference in *Ckip-1* 3′-UTR TG mice after simulated microgravity (Figures 6G,H). Consistent with the potential mechanism in mice, simulated microgravity induced downregulation of the CaKMM2-AMPK-PPARα-CPT1b axis-related protein expression in the hearts from rhesus monkeys after bed rest (Supplementary Figure S2). Collectively, these findings indicated that *Ckip-1* 3′-UTR overexpression remarkably alleviated lipid overload and improved cardiac function *via* the CaKMM2-AMPK-PPARα-CPT1b axis upon simulated microgravity (Figure 6I).

## Discussion

Under conditions of simulated microgravity, cardiac atrophy occurs gradually. Heart atrophic response to altered hemodynamics is a complex pathobiology process that is highly regulated by the transcription and post-transcription of the genes (Gao and Wang, 2020). However, the molecular basis of this heart disease remains to be elucidated yet. In this study, our results identified a 3′-UTR of *Ckip-1* mRNA that played a critical role in the simulated microgravity-induced cardiac atrophy. We found that the 3′-UTR and CDS region of *Ckip-1* mRNA showed diverse expression change in the hearts of mice and rhesus monkeys in response to simulated microgravity. Cardiac-specific overexpression of *Ckip-1* 3′-UTR dramatically induced decreased lipid deposition *via* activation of the CaKMM2-AMPK-PPARα-CPT1b signaling pathway, which inhibited heart atrophic development and left ventricular dysfunction after simulated microgravity. Taken together, this work provided compelling evidence that *Ckip-1* 3′-UTR will be a pivotal regulator of pathological cardiac remodeling during space flight.

The maturation of eukaryotic mRNAs underwent 5′ capping, introns splicing, and 3′-UTR processing. The 3′-UTRs were a vital class of long non-coding RNA, but their sequences were relatively conserved among various species (Mignone et al., 2002; Herzel et al., 2017). The genetic information encoded in the region of 3′-UTR was decrypted by a series of microRNAs and RNA-binding proteins; thereby, mRNA 3′-UTR acted as a principal platform to regulate mRNA metabolism, including nuclear-cytoplasmic shuttling, intracellular localization, translational activities, and mRNA stability (Mayr, 2017). Most pre-mRNAs were regulated by alternative polyadenylation, generating different mRNA isoforms with either a short or long 3′-UTR. Dynamic 3′-UTR length functioned as a novel mechanism to regulate cellular fate (Gruber and Zavolan, 2019). Moreover, 3′-UTR can regulate specific protein–protein interactions *via* establishing a nutritious niche (Ma and Mayr, 2018). The prevailing perspective was that 3′-UTR was regarded as a fraction of the canonical mRNA sequence. Previous studies had proved that 3′-UTRs in eukaryotic genes were expressed separately from the cognate mRNAs. Our recent study showed that the 3′-UTR region and coding sequence region of *Ckip-1* mRNA had unequal expression in heart failure and distinct localization in cardiomyocytes (Zhao et al., 2021). However, the role of *Ckip-1* 3′-UTR on cardiac atrophy was uncharacterized. In this study, our results showed the expression of *Ckip-1* 3′-UTR region was upregulated in the hearts from mice under 28 days of tail suspension and rhesus monkeys under 42 days of head-down bed rest, but *Ckip-1* CDS region was downregulated, and *Ckip-1* 3′-UTR was a crucial inhibitor of cardiac atrophic development.

Changes in gravitational loading in the space environment elicit an increased risk of heart disease. Ground-based analog models of space flight include hindlimb unloading by tail suspension, left ventricular assist device (LVAD)-induced mechanical unloading, and head-down tilt bed rest (Hill and Olson, 2008). Furthermore, it has been reported that extreme-duration swimming induced similar effects of cardiac atrophic growth to space flight because water pressure counteracted the gravitational force (MacNamara et al., 2021). Early studies showed that left ventricular mass of human volunteers decreased by 15% during 12 weeks of bed rest (Perhonen et al., 2001). In a recent NASA twins study, the astronaut was susceptible to cardiac injury in space flight. Similar to astronaut-related results, *Drosophila* showed that cardiac size and contraction were reduced in the International Space Station (ISS) compared with 1* g* (Walls et al., 2020). In a study, tail-suspended mice for 28 days caused a 5% ± 14% decrease in the heart weight-to-tibia length ratio (Liang et al., 2019). In summary, heart atrophic growth is the major challenge with manned deep-space expeditions and long-term residence in the International Space Station. Here, we demonstrated that *Ckip-1* 3′-UTR significantly protected against cardiac atrophy following simulated microgravity.

The heart is a highly energy-consuming tissue in maintaining sustained and rhythmic contraction. The cardiomyocytes were highly sensitive to changes in substrate utilization in physiological and pathological states. This metabolic flexibility contributed to optimal cardiac energy provision, but the main fuel switched from fatty acids to glucose during heart failure (Taegtmeyer et al., 2016). In simulated microgravity-induced cardiac remodeling, the atrophic heart in rats caused reduction in glucose consumption compared with control (Wang et al., 2020). H9C2 cardiomyocytes showed lower metabolic activity including reactive oxygen species (ROS) augmentation and mitochondrial superoxide anion increase in exposure to simulated microgravity for 96 h (Guarnieri et al., 2021). However, cardiac fatty acid metabolism under simulated weightlessness had not yet been explored intensively. We reported that simulated microgravity-induced cardiac lipid accumulation, and *Ckip-1* 3′-UTR overexpression remarkably abolished this phenotype.

We identified CaMKK2 as a critical target of *Ckip-1* 3′-UTR and restored fatty acid transport to the mitochondrion for ATP supply by activation the AMPK-PPAR-CPT1b signaling pathway, mitigating cardiac lipid deposition. Our results had demonstrated that *Ckip-1* 3′-UTR substantially alleviated cardiac atrophy after simulated microgravity. In conclusion, these results uncovered that *Ckip-1* 3′-UTR will be an effective non-coding RNA-based countermeasure for rescuing cardiac deconditioning under space flight.

## Data Availability

The original contributions presented in the study are included in the article/Supplementary Material, further inquiries can be directed to the corresponding authors.
