# Supplementary material for: Ckip-1 3′-UTR Attenuates Simulated Microgravity-Induced Cardiac Atrophy
Source: Front Cell Dev Biol. 2022 Feb 2;9:796902. doi: 10.3389/fcell.2021.796902 (PMC8847737; doi:10.3389/fcell.2021.796902)
Supplement: Supplementary file 1 [file DataSheet1.docx]

**SUPPLEMENTAL MATERIALS**

***Ckip-1* 3' UTR Attenuates Simulated Microgravity-Induced Cardiac Atrophy**

Yinglong Zhao^1,2#^, Guohui Zhong^1,3#^, Ruikai Du^1^, Dingsheng Zhao^1^, Jianwei Li^1^, Yuheng Li^1,3^, Wenjuan Xing^1, 3^, Xiaoyan Jin^1^, Wenjuan Zhang^4^, Weijia Sun^1^, Caizhi Liu^1^, Zizhong Liu^1^, Xinxin Yuan^1^, Guanghan Kan^1^, Xuan Han^1^, Qi Li^1^, Yan-Zhong Chang^2^*, Yingxian Li^1^* and Shukuan Ling^1^*

1. State Key Laboratory of Space Medicine Fundamentals and Application, China Astronaut Research and Training Center, Beijing, China.

2. Key Laboratory of Molecular and Cellular Biology of Ministry of Education, College of Life Science, Hebei Normal University, Shijiazhuang, China.

3. School of Aerospace Medicine, Fourth Military Medical University, Xi'an, China.

4. State Key Laboratory of Proteomics, National Center of Protein Sciences (Beijing), Beijing Institute of Lifeomics, Beijing, China.

# These authors contributed equally to this work.

* Corresponding author:

<Tel:+86>-133-11508172, E-mail: [sh2ling@126.com](mailto:sh2ling@126.com) (Shukuan Ling);

Tel: +86-135-52722709, E-mail: [yingxianli@aliyun.](mailto:yingxianli@aliyun.cn)com (Yingxian Li);

Tel: +86-311-80786311, E-mail: chang7676@163.com (Yan-zhong Chang).

**Supplemental Figures**


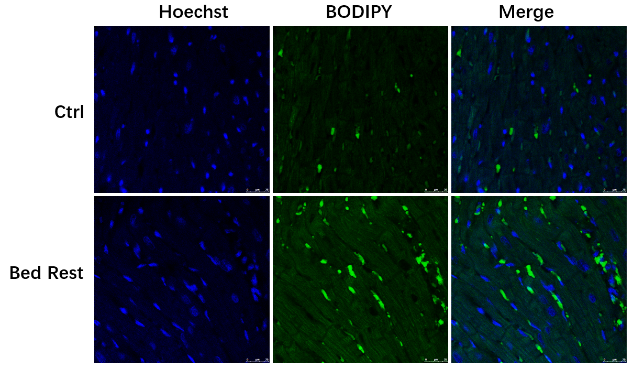


**Supplemental Figure 1. Representative images of lipid droplets visualized by BODIPY™ 493/503 in the hearts from rhesus monkeys in control group and the head-down bed rest morel group.** **Scale bar in sections, 25 µm.**


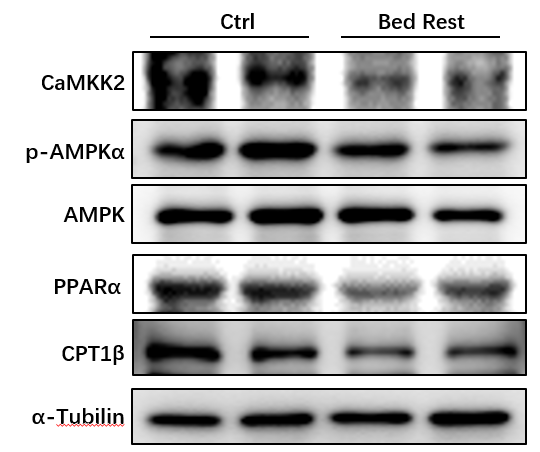


**Supplemental Figure 2. Western blot showing the data of CaMKK2, phosphorylated AMPK (T172), AMPK, PPARα, and CPT1b in the hearts from** **rhesus monkeys in control group and the head-down bed rest morel group.**
